# The association of seminal oxidation reduction potential with sperm parameters in patients with unexplained and male factor ınfertility

**DOI:** 10.1590/S1677-5538.IBJU.2019.0751

**Published:** 2020-11-18

**Authors:** Ozge Senem Yucel Cicek, Gozde Kaya, Begum Alyuruk, Emek Doger, Tugba Girisen, Serdar Filiz

**Affiliations:** 1 Kocaeli University Faculty of Medicine Department of Obstetrics and Gynecology IzmitKocaeli Turkey Department of Obstetrics and Gynecology, Kocaeli University, Faculty of Medicine, Izmit, Kocaeli, Turkey; 2 Kocaeli University Assisted Reproductive Technologies Clinic IzmitKocaeli Turkey Kocaeli University Assisted Reproductive Technologies Clinic; Izmit, Kocaeli, Turkey; 3 Kocaeli University Department of Histology and Embryology IzmitKocaeli Turkey Department of Histology and Embryology, Kocaeli University, Izmit, Kocaeli, Turkey

**Keywords:** Infertility, Male, Semen, Oxidative Stress

## Abstract

**Purpose:**

Understanding the effects of high oxidation reduction potential (ORP) levels on sperm parameters will help to identify patients with unexplained and male factor infertility who may have seminal oxidative stress and determine if ORP testing is needed. This study aimed to evaluate the association between seminal ORP and conventional sperm parameters.

**Materials and Methods:**

A total of 58 patients who provided a semen sample for simultaneous evaluation of sperm parameters and ORP between January and September 2019 were enrolled in this retrospective study. To identify normal and high ORP levels, a static ORP (sORP) cut-off value of 1.36mV/10^6^sperm/mL was used. Sperm parameters were compared between infertile men with normal sORP (control group, n=23) and high sORP values (study group, n=35).

**Results:**

Men with sORP values >1.36mV/10^6^sperm/mL had significantly lower total sperm count (TSC) (p <0.001), sperm concentration (p <0.001) and total motile sperm count (TMSC) (p <0.001). In addition, progressive motility (p=0.04) and fast forward progressive motility (p <0.001) were significantly lower in the study group. A negative correlation was found between sORP and TSC (r=-0.820, p <0.001), sperm concentration (r=-0.822, p <0.001), TMSC (r=-0.808, p <0.001) and progressive motility (r=-0.378, p=0.004). Non-progressive motility positively correlated with sORP (r=0.344, p=0.010).

**Conclusions:**

This study has shown that TSC, sperm concentration, progressive motility and TMSC are associated with seminal oxidative stress, indicated by a sORP cut-off of 1.36mV/10^6^sperm/mL. Presence of oligozoospermia, reduced progressive motilty or low TMSC in sperm analysis should raise the suspicion of oxidative stress and warrants seminal ROS testing.

## INTRODUCTION

Reactive oxygen species (ROS) are generated as by-products during normal metabolic events and play a crucial role in various cellular processes. The imbalance between ROS and antioxidant capacity due to excess production of ROS leads to oxidative stress (1). Oxidative stress contributes to the pathophysiology of many diseases, including cardiovascular, neurodegenerative diseases and cancer (2). Besides, ROS and oxidative stress have been implicated in the impairment of sperm functions.

ROS are normally present in human semen at low levels. The presence of leukocytes and immature, morphologically abnormal spermatozoa are the main causes of ROS in semen. Besides, exogenous stimuli including infections, environmental factors, and tobacco use can contribute to seminal ROS (3). Normal physiological levels of ROS are required for normal sperm functions, such as capacitation, acrosome reaction and sperm-oocyte fusion. However, spermatozoa are very sensitive to excess ROS due to their limited antioxidant capacity. Excessive ROS induces pathological processes in sperm cells, including lipid peroxidation and DNA damage, leading to sperm dysfunction (3).

Oxidative damage to sperm is a significant contributing factor in 30-80% of male factor infertility cases (1). Furthermore, higher ROS levels have been demonstrated in normozoospermic infertile men when compared to normozoospermic fertile controls, suggesting seminal oxidative stress might be a potential etiology in some unexplained cases of infertility (4).

Given the high incidence of seminal oxidative stress-related infertility, ROS screening during diagnostic evaluation of the male partner seems a reasonable approach. However, analysis of semen ROS status is not a standard procedure during fertility work-up, due to high cost and lack of a recognized, standardized measurement method (3). A new technology, the Male Infertility Oxidative System (MiOXSYS, Aytu BioScience Inc., Englewood, CO, USA) has recently become available. MiOXSYS is capable of calculating semen oxidation-reduction potential (ORP), a direct measure of oxidative stress and is a reliable method for assessing oxidative stress in semen and also easy to employ in clinical settings (5). However, there is no consensus on which patients should be tested for seminal ROS (6).

This study aimed to evaluate the association of normal and elevated ORP levels with conventional sperm parameters in patients with unexplained and male factor infertility. A further aim was to investigate the correlations between seminal ORP and sperm parameters. We hypothesized that by establishing the effects of high ORP levels on sperm parameters, it would be possible to identify patients likely to have seminal oxidative stress, based on routine semen analysis and determine if further ORP testing is needed.

## MATERIALS AND METHODS

### Study design and participants

This was a retrospective cohort study conducted at Kocaeli University Faculty of Medicine Assisted Reproductive Technologies (ART) Clinic, Kocaeli, Turkey. The Institutional Review Board of Kocaeli University Faculty of Medicine approved the study (approval number: GOKAEK-2019/17.08 2019/277, date: 17.10.2019).

The study population included men attending the ART clinic for infertility evaluation between January and September 2019. Men providing a semen sample for simultaneous evaluation of sperm parameters and ORP were included. All patients underwent andrological evaluation including medical history, physical examination and sperm analysis and their partners were assessed for female infertility factors including tubal occlusion, and evidence of uterine pathologies and/or ovulatory disorders. Men with a diagnosis of a sexually transmitted disease, a history of chemotherapy or radiotherapy or a loss of sample during collection were excluded from the study. Men using antioxidant supplementation for any reason were also excluded.

Between January 2019 and September 2019, a total of 58 men were eligible for the study. Of these, 23 were normozoospermic and 35 had altered semen characteristics according to the World Health Organization (WHO) 2010 criteria (7). Four normozoospermic patients having a round cell concentration exceeding 1×10^6^per mL and one normozoospermic patient with a varicocele evident during a Valsalva maneuver were considered as having male factor infertility. The remaining 18 normozoospermic patients had unexplained infertility (defined as normozoospermia and absence of a female factor infertility).

To identify normal and high ORP levels, a static ORP (sORP) cut-off value of 1.36 mV/10^6^sperm/mL, as established by Agarwal et al. was used (8). Men with a sORP value of >1.36mV/10^6^ sperm/mL were deemed to have elevated sORP while those with a value of ≤1.36mV/10^6^ sperm/mL were deemed to have normal sORP. Sperm parameters were compared between infertile men with normal sORP (control group) and high sORP values (study group).

### Semen analysis

Semen samples were obtained by masturbation after three to five days of sexual abstinence. Men were asked to report any loss of the sample during collection. Sample containers were kept in an incubator at 37°C for 30 minutes. After liquefaction, semen analyses were performed according to the 5th Edition of the WHO laboratory manual for the examination and processing of human semen (7). Motility was graded as progressive motility, non-progressive motility, and immotility. Progressive motility was further graded as (A) fast forward progressive and (B) slow forward progressive, according to the 4th Edition of WHO laboratory manual (9). Total motile sperm count (TMSC) was calculated by multiplying the semen volume (mL) by sperm concentration (10^6^sperm/mL) and the percentage of A+B motility divided by 100% (10). Evaluation of at least 200 spermatozoa in a total of at least five fields in each replicate was performed to avoid sampling error.

### ORP analysis

sORP measurement using the MiOXSYS system was performed to analyze oxidative stress in semen. After liquefaction, 30uL of unprocessed semen sample was applied to the MiOXSYS sensor and the sample was processed automatically. After analysis, the sORP value was displayed in millivolts (mV). The norming of sORP values to sperm concentration was performed and normed sORP values were expressed in mV/10^6^sperm/mL.

### Statistical Analysis

All statistical analyses were performed using the Statistical Package for Social Sciences (SPSS) version 21.0 (IBM Corp, Armonk, NY, USA). Kolmogorov-Smirnov tests were used for the assessment of the normality of data distribution. Continuous variables with normal distribution were expressed as mean±standard deviation (SD) and continuous variables with non-parametric distribution were expressed as median and interquartile range (IQR). The comparison of numerical variables was performed using Student t-test for continuous variables with normal distribution and Mann-Whitney U test for continuous variables without normal distribution. Depending on data distribution, Pearson or Spearman's Rho correlation coefficients were used to test the association between numerical variables. A p-value <0.05 was considered statistically significant.

## RESULTS

During the study period, 58 men presented who were eligible for the study. Of these, 23 (39%) had normal sORP values and constituted the control group and 35 (61%) had high sORP values and made up the study group. Baseline characteristics and comparison of sperm parameters between the control and study groups are presented in Table-1. There was no significant difference between men with normal and high sORP values regarding age and body mass index (p=0.107 and p=0.962, respectively). The control group had a median sORP value of 0.38 (0.23-0.76) mV/10^6^sperm/mL whereas the study group had a median sORP of 6.71 (2.35-21.17) mV/10^6^sperm/mL.

**Table 1:** Baseline characteristics and comparison of sperm parameters between infertile men with normal and high sORP values.

Parameters	sORP value		p value
	≤1.36 (n=23)	>1.36 (n=35)	
Age (years)	37.5±5.2	35.2±5.3	0.107*
BMI (kg/m^2^)	26.1 (24.4-27.7)	25.8 (23.8-29.3)	0.962**
Semen volume (mL)	3.4 (1.8-4.2)	2.6 (1.8-3.5)	0.399**
Total sperm count (x10^6^)	221.0 (91.8-324.0)	9.35 (2.88-81.60)	<0.001**
Sperm concentration (x10^6^/mL)	60.0 (34.0-140.0)	10.0 (1.7-27.7)	<0.001**
TMSC (x10^6^)	60.0 (30.6-163.4)	3.1 (0.8-29.9)	<0.001**
Total motility (%)	48.6±15	43.6±18	0.302*
Progressive motility (%)	39.8±16	30.9±14	0.040*
Fast forward progressive motility (%)	5.0 (0-5)	0 (0-0)	<0.001**
Non-progressive motility (%)	8.7±4	12.6±9	0.081*
Immotility (%)	49.1±18	56.3±18	0.163*
Round cell (x10^6^)	0.10 (0.1-0.6)	0.20 (0.1-0.4)	0.689**

Variables are given as median (interquartile range) or mean ± SD.

sORP, static oxidation reduction potential; BMI, body mass index; TMSC, total motile sperm count

* Student's t test

** Mann Whitney U test

When patients were grouped according to etiology of infertility; four of 18 (22%) patients with unexplained infertility had elevated seminal sORP and 31 out of 40 (77.5%) patients with male factor infertility had elevated sORP values. Median sORP value of unexplained infertility patients was 0.38 (0.28-1.07) mV/10^6^sperm/mL whereas male factor infertility patients had a median sORP value of 5.52 (1.42-19.32) mV/10^6^sperm/mL. Male factor infertility patients had significantly higher seminal sORP values compared to unexplained infertility patients (p <0.001).

When patients were grouped according to WHO semen analysis reference values, normozoospermic patients had significantly lower sORP values compared to non-normozoospermic ones. Normozoospermic men had a median sORP value of 0.420 (0.340-1.480) mV/10^6^sperm/mL whereas men with altered semen parameters had a median sORP value of 6.50 (1.84-21.17) mV/10^6^sperm/mL (p <0.001).

Men with sORP values >1.36mV/10^6^sperm/mL had significantly lower total sperm count (TSC) (p <0.001), sperm concentration (p <0.001) and TMSC (p <0.001). In addition, progressive motility and fast forward progressive motility were significantly reduced in the study group (p=0.04 and p <0.001, respectively).

Semen volume (p=0.399), total motility (p=0.302), non-progressive motility (p=0.081), immotility (p=0.163) and round cell numbers (p=0.689) did not differ significantly between the study and control groups. The mean total motility was above the WHO lower reference limit (40%) in both the control and study groups.

**Figure 1:**
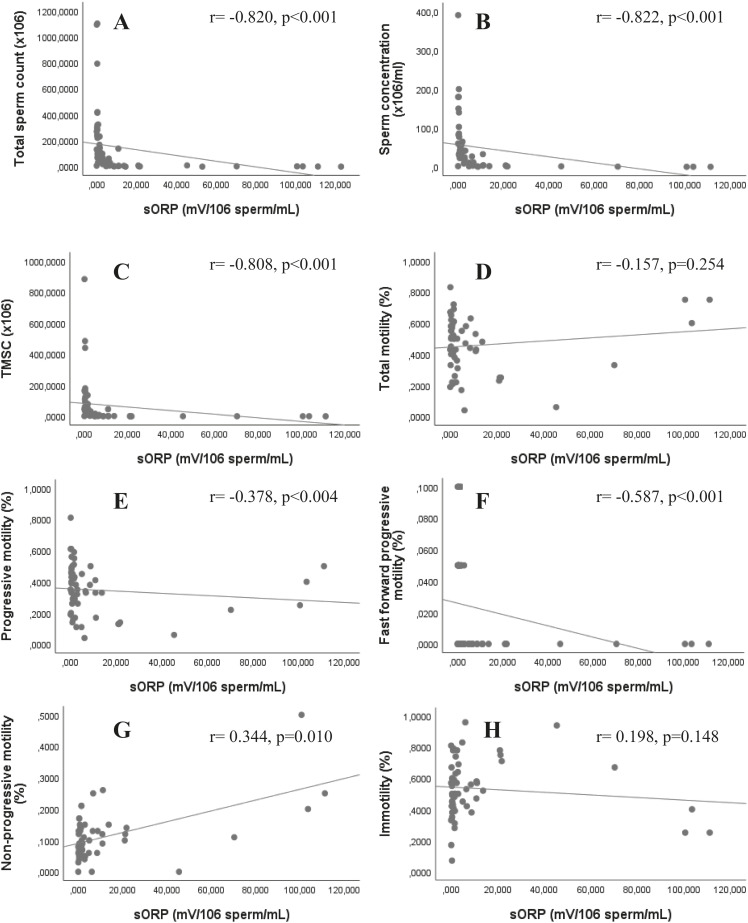
Correlation of static oxidation reduction potential (sORP) with sperm parameters. A: total sperm count, B: concentration, C: total motile sperm count (TMSC), D: total motility, E: progressive motility, F: fast forward progressive motility, G: non progressive motility, H: immotility. sORP negatively correlates with total sperm count, sperm concentation, TMSC, progressive and fast forward progressive motility.

Correlations between sperm parameters and sORP are presented in Figure-1A-H. Strong negative correlations were found between sORP and TSC (r=-0.820, p <0.001), sperm concentration (r=-0.822, p <0.001) and TMSC (r=-0.808, p <0.001). In addition, there was a significant negative correlation between sORP and progressive motility (r=-0.378, p=0.004) and fast forward progressive motility (r=-0.587, p <0.001). However, no correlation was found between sORP and total motility (r=-0.157, p=0.254) and immotility (r=0.198, p=0.148). Non-progressive motility positively correlated with sORP (r=0.344, p=0.010). There was no correlation between sORP and semen volume (r=-0.121, p=0.364) and round cell numbers (r=0.010, p=0.941).

## DISCUSSION

ORP, measured by the MiOXSYS system, is a direct measure of oxidative stress in semen and evaluates the balance between all oxidants and all available antioxidants in the sample (5). In this retrospective study, the association of seminal ORP levels with routinely assessed sperm parameters was investigated. This study showed that men with high ORP levels had impaired sperm parameters, including count, concentration, and motility compared to men with normal seminal ORP.

Oxidative stress has been shown to play a direct role in the etiology of male infertility (3). Several studies reported a high incidence of seminal oxidative stress in infertile men (1, 6, 11). The role of oxidative stress in men with unexplained infertility is less clear. In our cohort, high ORP values were found in 22% of men with unexplained infertility. This finding is consistent with a previous study that demonstrated higher seminal ROS in men with unexplained infertility compared to healthy controls (4). Besides, Shekarriz et al. investigated seminal ROS formation in healthy men and showed that ROS formation was negative in all healthy donors (11).

Recently, a new entity, male oxidative stress infertility (MOSI), has been proposed to define infertile men with seminal oxidative stress and the authors suggested ORP as the clinical biomarker of MOSI (12). Although, ORP measurement by the MiOXSYS system is a promising method and has been shown to be easy, quick and reliable (5), its use in the evaluation of male infertility is not common. Identification of routine semen analysis parameters with a strong correlation with high ORP values might help diagnose patients with MOSI.

Correlation analysis of ORP levels with TSC showed a strong negative correlation. Sperm concentration also negatively correlated with sORP. Our findings confirm previous results (5, 8, 13). However, in our study, 13 out of 34 infertile patients with normal TSC and concentration also had elevated sORP values, suggesting normal TSC and concentration do not rule out high ROS.

A prospective cohort study evaluating the effects of oxidative stress on sperm plasma membrane integrity found that hydrogen peroxide caused a dose-dependent decrease in sperm motility (14). Agarwal et al. found higher sORP in patients with poor total motility and a negative correlation of sORP with motility in their earlier studies describing ORP measurement protocol using the MiOXSYS system and in which they established a reference value for sORP (5, 8). However, they did not grade sperm motility. Another study reported a negative correlation between sORP and progressive motility (15). Consistent with these reports, we found reduced motility in patients with elevated sORP values compared to patients with normal sORP values. We also found a significant negative correlation between sORP value and progressive sperm motility and fast forward progressive motility and a positive correlation between sORP and non-progressive motility, suggesting higher ROS levels impair progressive motility rather than affecting total motility or causing immotility. Since progressive motility is critical for normal sperm function and progressive motility rates are associated with pregnancy rates (16), measurement of ROS in patients with poor progressive motility, even if they have normal total motility, appears to be reasonable.

The relationship between oxidative stress and sperm motility can be explained by the pathological processes which occur in spermatozoa in the presence of ROS. Excessive levels of ROS initiate a process leading to lipid peroxidation. As the sperm plasma membrane is rich in lipid components, it is a potential target for oxidative stress (17). It has been shown that lipid peroxidation causes loss of membrane fluidity and function and subsequently results in impaired sperm motility (18). However, antioxidants have been shown to reduce oxidative stress and improve sperm motility, enabling oxidative stress-mediated motility loss a treatable cause of male infertility (1).

Our analysis showed that men with higher sORP had lower TMSC. In addition, there was a strong negative correlation between sORP and TMSC. The 5th Edition of the WHO manual classifies the quality of semen based on three sperm parameters: number; motility; and morphology (7). Abnormal semen analysis is described according to deviations from the reference range for each parameter. However, many authors have argued that TMSC is a better measure of male factor infertility. A prospective cohort study by Hamilton et al. showed that TMSC has prognostic value for natural ongoing pregnancy rates and suggested using TMSC as the method of choice to express the severity of male infertility (10). Besides, TMSC was found to be a better predictor than the WHO manual reference values for the outcomes of intracytoplasmic sperm injection (ICSI) cycles (19). Assuming that TMSC is a valid parameter for describing sperm quality, our results suggest excessive ROS has a detrimental effect on sperm quality. In addition, low TMSC may be a consequence of high oxidative stress and the prognostic value of TMSC, both for natural pregnancy and ICSI cycles, justifies ROS testing in patients with low TMSC.

Spermatozoa are protected from oxidative stress by several antioxidants. Once oxidative stress is diagnosed as the underlying cause of male infertility, there are available options for treatment. Apart from the identification of possible causes of oxidative stress, lifestyle modifications and avoiding environmental exposure, oral antioxidants are an effective treatment option with low cost and relatively minor side effects (1). Various studies suggest oral antioxidants can reduce seminal ROS levels (20, 21). Moreover, a recent Cochrane review has shown that antioxidant supplementation may lead to increased clinical pregnancy and live birth rates in subfertile men (22). On the other hand, overtreatment with antioxidants may tip the system towards the reduced ROS status which is also harmful. Therefore, the assessment of seminal ROS is essential prior to antioxidant treatment (23).

Our research has some limitations. The first is the retrospective design of the study. The second is we did not evaluate the correlation between sperm morphology and sORP due to high interobserver variability in the assessment of morphology and low predictive value of morphology for pregnancy success (24). Importantly, several reports found a negative correlation between oxidative stress and sperm morphology (13, 25, 26). Further large, prospective studies should focus on the relationship between sORP values and ART cycle outcomes.

## CONCLUSIONS

Semen analysis is the initial step in andrological evaluation. However, seminal ROS measurement is not carried out routinely as part of male infertility workup. Given that elevated ROS levels have been demonstrated in patients with male factor infertility and also unexplained infertility, seminal ROS evaluation may need to be adopted more widely during male fertility assessment.

It is essential to diagnose oxidative stress-related infertility since there are effective treatment options leading to improved pregnancy rates. As it is not feasible to perform seminal ROS analysis in every patient, identification of sperm parameters associated with elevated ROS levels will be of benefit. Our analysis has shown that total sperm count, sperm concentration, progressive motility, and TMSC are related to seminal ROS. The presence of oligozoospermia, reduced progressive motility or low TMSC in a sperm analysis should raise the suspicion of elevated ROS and warrants seminal ROS testing.

## ABBREVIATIONS

ART: = Assisted Reproductive Technologies
ICSI: = Intracytoplasmic Sperm Injection
IQR: = interquartile range
MOSI: = male oxidative stress infertility
ORP: = oxidation reduction potential
ROS: = reactive oxygen species
SD: = standard deviation
sORP: = static oxidation reduction potential
TSC: = total sperm count
TMSC: = total motile sperm count
WHO: = World Health Organization
